# Vaginal progesterone pessaries for pregnant women with a previous preterm birth to prevent neonatal respiratory distress syndrome (the PROGRESS Study): A multicentre, randomised, placebo-controlled trial

**DOI:** 10.1371/journal.pmed.1002390

**Published:** 2017-09-26

**Authors:** Caroline A. Crowther, Pat Ashwood, Andrew J. McPhee, Vicki Flenady, Thach Tran, Jodie M. Dodd, Jeffrey S. Robinson, for the PROGRESS Study Group

**Affiliations:** 1 Liggins Institute, University of Auckland, Auckland, New Zealand; 2 School of Medicine, The University of Adelaide, Adelaide, Australia; 3 Robinson Research Institute, University of Adelaide, Adelaide, Australia; 4 Department of Neonatal Medicine, Women’s and Children’s Hospital, Adelaide, Australia; 5 Mater Research Institute, Faculty of Medicine, University of Queensland, Australia; 6 Osteoporosis and Bone Biology, Garvan Institute of Medical Research, Sydney, Australia; University of Manchester, UNITED KINGDOM

## Abstract

**Background:**

Neonatal respiratory distress syndrome, as a consequence of preterm birth, is a major cause of early mortality and morbidity. The withdrawal of progesterone, either actual or functional, is thought to be an antecedent to the onset of labour. There remains limited information on clinically relevant health outcomes as to whether vaginal progesterone may be of benefit for pregnant women with a history of a previous preterm birth, who are at high risk of a recurrence. Our primary aim was to assess whether the use of vaginal progesterone pessaries in women with a history of previous spontaneous preterm birth reduced the risk and severity of respiratory distress syndrome in their infants, with secondary aims of examining the effects on other neonatal morbidities and maternal health and assessing the adverse effects of treatment.

**Methods:**

Women with a live singleton or twin pregnancy between 18 to <24 weeks’ gestation and a history of prior preterm birth at less than 37 weeks’ gestation in the preceding pregnancy, where labour occurred spontaneously or in association with cervical incompetence or following preterm prelabour rupture of the membranes, were eligible. Women were recruited from 39 Australian, New Zealand, and Canadian maternity hospitals and assigned by randomisation to vaginal progesterone pessaries (equivalent to 100 mg vaginal progesterone) (*n* = 398) or placebo (*n* = 389). Participants and investigators were masked to the treatment allocation. The primary outcome was respiratory distress syndrome and severity. Secondary outcomes were other respiratory morbidities; other adverse neonatal outcomes; adverse outcomes for the woman, especially related to preterm birth; and side effects of progesterone treatment. Data were analysed for all the 787 women (100%) randomised and their 799 infants.

**Findings:**

Most women used their allocated study treatment (740 women, 94.0%), with median use similar for both study groups (51.0 days, interquartile range [IQR] 28.0–69.0, in the progesterone group versus 52.0 days, IQR 27.0–76.0, in the placebo group). The incidence of respiratory distress syndrome was similar in both study groups—10.5% (42/402) in the progesterone group and 10.6% (41/388) in the placebo group (adjusted relative risk [RR] 0.98, 95% confidence interval [CI] 0.64–1.49, *p* = 0.912)—as was the severity of any neonatal respiratory disease (adjusted treatment effect 1.02, 95% CI 0.69–1.53, *p* = 0.905). No differences were seen between study groups for other respiratory morbidities and adverse infant outcomes, including serious infant composite outcome (155/406 [38.2%] in the progesterone group and 152/393 [38.7%] in the placebo group, adjusted RR 0.98, 95% CI 0.82–1.17, *p* = 0.798). The proportion of infants born before 37 weeks’ gestation was similar in both study groups (148/406 [36.5%] in the progesterone group and 146/393 [37.2%] in the placebo group, adjusted RR 0.97, 95% CI 0.81–1.17, *p* = 0.765). A similar proportion of women in both study groups had maternal morbidities, especially those related to preterm birth, or experienced side effects of treatment. In 9.9% (39/394) of the women in the progesterone group and 7.3% (28/382) of the women in the placebo group, treatment was stopped because of side effects (adjusted RR 1.35, 95% CI 0.85–2.15, *p* = 0.204). The main limitation of the study was that almost 9% of the women did not start the medication or forgot to use it 3 or more times a week.

**Conclusions:**

Our results do not support the use of vaginal progesterone pessaries in women with a history of a previous spontaneous preterm birth to reduce the risk of neonatal respiratory distress syndrome or other neonatal and maternal morbidities related to preterm birth. Individual participant data meta-analysis of the relevant trials may identify specific women for whom vaginal progesterone might be of benefit.

**Trial registration:**

Current Clinical Trials ISRCTN20269066.

## Abstract 2

In a randomized controlled trial, Caroline Crowther and colleagues examine the effect of vaginal progesterone pessaries for pregnant women to prevent neonatal respiratory distress syndrome.

## Author summary

**Why was the study done?:**

- Prevention of preterm birth remains a global challenge. Vaginal progesterone has been suggested to reduce the incidence of preterm birth in women at risk, but there has been limited assessment of the effects on relevant neonatal morbidity.
- The aim of our trial was to assess whether vaginal progesterone pessaries in pregnant women with a history of previous spontaneous preterm birth reduced the risk and severity of respiratory distress syndrome, other neonatal morbidities, and maternal health outcomes and if there were any side effects of treatment.

**What did the researchers do and find?:**

- Seven hundred and eighty-seven women were randomly allocated to receive vaginal progesterone or placebo from between 20 to 24 weeks’ to 34 weeks’ gestation.
- The rate of respiratory distress syndrome amongst infants in both treatment groups was similar, as were the outcomes for other respiratory measures and adverse outcomes.
- Maternal health outcomes and side effects of treatment were also similar between treatment groups.

**What do these findings mean?:**

- Vaginal progesterone pessaries did not reduce the risk of respiratory distress syndrome compared to placebo vaginal pessaries.
- Vaginal progesterone pessaries cannot be recommended in women with a history of previous preterm birth to reduce infant or maternal morbidity associated with any recurrence.
- These results provide robust evidence for health practitioners and consumers to make informed clinical decisions.

## Introduction

The prevention of preterm birth remains a global challenge [1]. Women who have had a previous preterm birth have over twice the risk of giving birth preterm in a subsequent pregnancy [2,3,4]. Babies born preterm are at increased risk of respiratory distress syndrome as a result of immature lung development, and this is a major cause of their early neonatal mortality and morbidity [5] as well as long-term morbidity [6,7].

Progesterone has an important role in uterine quiescence [8,9] and is essential for the maintenance of pregnancy through multiple and complex mechanisms [10,11,12].

An initial systematic review of studies from the 1960s showed that use of progesterone may prevent preterm birth [13]. Over the last decade, there has been renewed interest in the use of vaginal progesterone in pregnancy to prevent recurrence of preterm birth, with several published trials included in the Cochrane systematic review [14]. Some trials suggest that use of vaginal progesterone reduces the risk of preterm birth [15], whilst others do not [16]. This has led to considerable debate and differences in clinical practice recommendations [17,18,19,20].

At the time of planning our trial, there were 2 published clinical trials with relatively small sample sizes that included women with a previous history of preterm birth, and these studies had shown a reduction in preterm birth with the use of both natural vaginal progesterone [15] and intramuscular injection of 17 OH progesterone, a synthetic progestogen [21]. However, intramuscular 17 alpha-hydroxyprogesterone caproate is not available for use in some countries, including Australia and New Zealand. Whilst a reduction in preterm birth may seem beneficial, prolongation of gestation may not lead to health benefits, so it also is important to know the effects on neonatal morbidities, such as respiratory distress syndrome and its sequelae, and on maternal health outcomes.

The primary aim of the PROGRESS randomised, placebo-controlled trial was therefore to assess whether the use of vaginal progesterone pessaries in pregnant women with a history of previous spontaneous preterm birth reduced the risk and severity of respiratory distress syndrome, thus improving the infant’s health. The secondary aims were to examine the effects on other respiratory outcomes; other neonatal morbidities; and maternal health outcomes, especially those related to preterm birth; and to assess any side effects of treatment.

## Methods

### Design and participants

We conducted a multicentre, placebo-blinded, randomised controlled trial at 39 Australian, New Zealand, and Canadian maternity hospitals. This study is reported as per CONSORT guidelines (S1 Text). The study was approved by the Children’s Youth and Women’s Health Services Human Research Ethics Committee at the Women’s and Children’s Hospital, Adelaide, Australia (approval record number HREC 2006015), and by the ethics committee at each of the 39 collaborating centres (32 in Australia, 5 in New Zealand, and 2 in Canada).

Women were eligible if they had a live singleton or twin pregnancy between 18 and <24 weeks’ gestation and a history of prior preterm birth (either vaginal birth or caesarean birth) at greater than 20 weeks’ gestation and less than 37 weeks’ gestation in their preceding pregnancy where the onset of labour occurred spontaneously or in association with cervical incompetence or following preterm prelabour rupture of membranes. If the women had received progesterone therapy prior to 16 weeks’ gestation, they remained eligible. The protocol for this study has been published [22] (S2 Text).

Women were ineligible if their preceding preterm birth at less than 37 weeks’ gestation was associated with placental abruption or placenta praevia, if it was a multiple pregnancy, or if there had been an iatrogenic decision for early birth, for example, related to fetal distress or preeclampsia.

Women were ineligible if their current pregnancy, at consideration for trial entry, was associated with active vaginal bleeding requiring hospital admission at 18 weeks’ gestation or more, preterm prelabour rupture of membranes, active labour (defined as the presence of uterine activity and cervical dilatation greater than 3 cm), known lethal fetal anomaly or fetal demise, progesterone treatment after 16 weeks’ gestation, or any contraindication to continuation of the pregnancy, such as chorioamnionitis requiring delivery, or contraindication to progesterone therapy (known active liver disease, active or hormone-related thrombophlebitis or thromboembolic disorder, or breast or genital malignancy). The PROGRESS Study protocol did not include the need for cervical length measurement at trial entry or during the pregnancy. The clinician responsible for care of the participant decided whether cervical length screening was undertaken.

The study was approved by the Children’s Youth and Women’s Health Services Human Research Ethics Committee at the Women’s and Children’s Hospital, Adelaide, Australia, and by the ethics committee at each of the 39 collaborating centres (33 in Australia, 4 in New Zealand and 2 in Canada).

Eligible women were provided with written information about the study in the antenatal clinic, counselled by 1 member of the research team, and asked if they would participate. Recruitment started in February 2006 and was completed in September 2012.

### Randomisation

Women who gave written informed consent were randomly assigned to either ‘progesterone’ or 'placebo’ using a central telephone randomisation service. The randomisation schedule, prepared by an investigator not involved with clinical care, used balanced variable blocks with stratification by plurality of the pregnancy (singleton versus twin versus triplet) and collaborating centre. Participants, staff, and investigators were masked to study group allocation, and treatment packs appeared identical. The baseline information collected included maternal age, parity, ethnicity, body mass index, plurality, gestational age at trial entry, gestational age, and reason for the previous preterm birth.

### Intervention and outcomes

#### Progesterone group and placebo group

Women randomised to the progesterone and placebo groups were allocated a study number that corresponded to a treatment pack containing the allocated study treatment.

Depending on the study treatment allocation, the treatment packs contained either a 14-week supply of progesterone pessaries (equivalent to 100 mg vaginal progesterone as active substance in hard fat) or similar-appearing placebo pessaries (in hard fat) bought for the study from Orion Laboratories, Western Australia. The manufacturer of the pessaries had no other involvement in the study. Women were asked to self-administer a vaginal pessary each evening from 20 weeks’ gestation, or from randomisation if this occurred after 20 weeks’ gestation, until birth or 34 weeks’ gestation, whichever occurred first. The maximum number of days treatment could be used for was 98 days.

Women were reviewed in the antenatal clinic by the practitioner responsible for their care. Women who presented with preterm prelabour rupture of the membranes after trial entry were advised to discontinue using the vaginal pessaries to reduce the risk of introducing infection. In the event of the development of serious depression or a medical condition that may have been aggravated by fluid retention (asthma, epilepsy, migraine, known cardiac dysfunction, or known renal dysfunction), the clinician was to advise the woman to cease using the trial medication if he or she felt it would be in the woman’s best interests to do so.

At 34 weeks’ gestation, women were asked to complete a questionnaire that assessed health-related quality of life [23], anxiety [24], and depression [25] and asked about any side effects they may have experienced and their compliance with the treatment protocol. After birth, information relating to birth, maternal and infant health, and care was collected from the woman's and infant's case notes by trained research assistants.

### Study outcomes

#### Primary outcome

The primary outcome was the incidence of neonatal respiratory distress syndrome (defined as increasing respiratory distress or oxygen requirement or the need for respiratory support from the first 6 hours of life) and severity of neonatal respiratory disease (defined as mild = mean airway pressure [MAP] < 7 cm H_2_O and/or fractional inspired oxygen [FiO_2_] < 0.4; moderate = MAP 7–9.9 cm H_2_O and/or FiO_2_ 0.40–0.79; severe = MAP ≥ 10 cm H_2_O, and/or FiO_2_ ≥ 0.80 with need for ventilation).

#### Secondary outcomes for the child

The secondary outcomes for the child were as follows:

1. other respiratory measures, which included the need for and duration of oxygen therapy (including highest FiO_2_ [%] within 12 hours of birth), need for and duration of mechanical ventilation (including maximum peak pressure [cm H_2_O] within 12 hours of birth), need for surfactant therapy, nitric oxide for respiratory support, air leak syndrome, and chronic lung disease (defined as the need for any respiratory support, supplemental oxygen, or intermittent positive pressure ventilation or continuous positive airways pressure for a chronic pulmonary disorder on the day the baby reached 36 weeks’ postmenstrual age for infants born before 32 weeks’ gestation, or continued oxygen requirement at 28 days of age for infants born after 36 weeks’ gestation) and
2. a composite adverse outcome for the infant that included 1 or more of the following: preterm birth (defined as birth at less than 37 weeks’ gestation), perinatal mortality (defined as either a stillbirth [intrauterine fetal death after trial entry and prior to birth] or infant death [death of a live-born infant prior to hospital discharge] and excluding lethal congenital anomalies), severe respiratory disease, chronic lung disease, Apgar score < 4 at 5 minutes of age, birth weight less than the third centile for gestational age at birth and infant sex, intraventricular haemorrhage on early cranial ultrasound, periventricular leucomalacia on later cranial ultrasound, inotropic support for the treatment of patent ductus arteriosus, proven necrotising enterocolitis, proven systemic infection within 48 hours of birth treated with antibiotics, and retinopathy of prematurity.

#### Secondary study outcomes for the mother

The secondary study outcomes for the mother were as follows:

1. significant health outcomes, particularly related to preterm birth, such as use of tocolytic therapy or antenatal corticosteroid therapy, defined by 1 or more of the following: maternal death, antepartum haemorrhage, pre-eclampsia, preterm prelabour rupture of membranes, prelabour ruptured membranes at or near term (defined as prelabour rupture of membranes after 36 weeks’ gestation), chorioamnionitis requiring antibiotic use during labour, postpartum haemorrhage, or antibiotic use after birth;
2. length of any antenatal hospital stay or postnatal stay and psychological health (assessed by quality of life [23], anxiety [24], and depression [25]); and
3. side effects of progesterone supplementation (including headache, nausea, pain and discomfort, breast tenderness, and coughing) and if any of them were sufficient to stop treatment.

### Statistical methods

Primary analyses were performed on an intention-to-treat basis, according to the study group allocated at randomisation. As prespecified, unadjusted analyses were performed and then adjusted for the potential confounders of gestational age at randomisation, gestational age of the previous preterm birth, and reason for the previous preterm birth.

Binary outcomes were analysed using log binomial regression, with treatment effects expressed as relative risk (RR) with 95% confidence interval (CI), or Fisher’s exact tests with no adjustment for covariates in the case of rare outcomes. Outcomes measured on a continuous scale were analysed using linear regression, with treatment effects expressed as differences in means. Count outcomes were analysed using Poisson regression or negative binomial regression where overdispersion was present, with treatment effects expressed as ratios of means. Ordinal outcomes were analysed using proportional odds models, with treatment effects expressed as odds ratios of higher severity. For infant outcomes, clustering due to multiple births was taken into account using generalised estimating equations. Statistical significance was assessed at the 2-sided *p* < 0.05 level, and no adjustment was made for multiple comparisons. No adjustments were made for the 2 primary outcomes, as they were considered strongly related and expected to provide complementary information [26]. All analyses followed a prespecified statistical analysis plan and were performed using SAS software version 9.3 (SAS Institute, Cary, North Carolina, United States).

#### Sample size

We originally estimated that a sample size of 984 women would be able to show a 40% reduction in neonatal respiratory distress syndrome from 15% to 9% with progesterone supplementation (5% level of significance, 2-tailed alpha, 80% power, 4% loss to follow-up) based upon data from a randomised trial with similar eligibility profile when this trial commenced [21]. In 2009, because of slower than anticipated accrual, we applied for additional funding to complete the study. At this time, the Trial Steering Group asked the following questions of an independent review: (1) ‘Should recruitment stop (because of a significant result or futility)?’ (2) ‘Should we continue recruiting to reach our previous sample size?’ and (3) ‘Does the sample size need refining based on the interim assessment?’ The Trial Steering Group did not see the interim data or the analyses. The independent review undertaken, masked to treatment group, made the following recommendations to the Trial Steering Group: to continue recruitment and to reduce the sample size to 784 women.

## Results

### Baseline information

Of an estimated 1,919 eligible women able to be approached by the research team between February 2006 and September 2012, a total of 787 (41%) women consented to be enrolled in the study. Reasons for eligible women declining to participate included ‘not interested in research’ (25%), ‘concerned about side effects and risks of use of drugs in pregnancy’ (15%), ‘no reason given’ (13%), ‘did not like the need to use vaginal pessaries’ (9%), ‘too busy’ (8%), ‘did not consider themselves to be at risk of preterm birth’ (6%), ‘partner declined to let them participate’ (5), and ‘other’ (19%).

**Fig 1:**
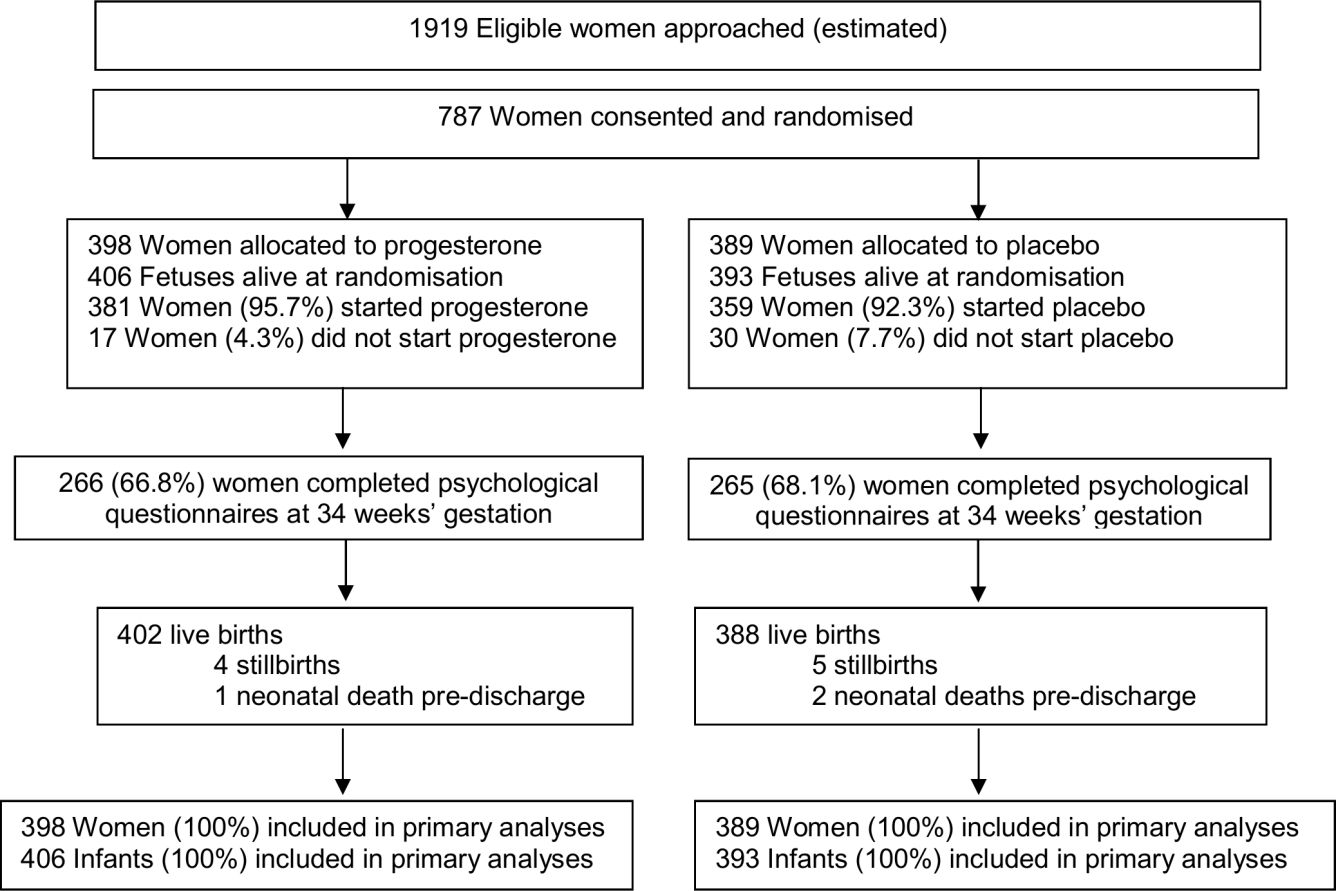
Consolidated Standards of Reporting Trials (CONSORT) flow diagram of participants in the study.

Of the 787 women recruited, 398 (50.6%) were randomised to the progesterone group, and 389 (49.4%) to the placebo group. There were no losses to follow-up, with clinical outcomes to primary hospital discharge after birth available for all 787 (100%) women and their 799 infants (Fig 1).

The 2 study groups were similar at the time of study entry for maternal demographics and key variables including gestational age, the reason for the preterm birth in the preceding pregnancy, and the gestational age at which that birth occurred (Table 1). The majority of participants had a singleton pregnancy, with less than 2% having a twin pregnancy (Table 1). Almost all women recruited in both study groups used their allocated study treatment (381 [95.7%] in the progesterone group and 359 [92.3%] in the placebo group), with similar median days of use in both study groups (51.0 days [interquartile range (IQR) 28.0–69.0] in the progesterone group versus 52.0 days [IQR 27.0–76.0] in the placebo group) (Table 1).

**Table 1:** Comparability of randomised study groups at trial entry and use of study treatment.

Characteristic	Progesterone (*n* = 398)	Placebo (*n* = 389)
Maternal age (years)*	30.3 (5.5)	30.3 (5.6)
Public patient	343 (86.2)	347 (89.2)
Ethnicity^‡^		
White	286 (71.9)	289 (74.3)
Asian	43 (10.8)	41 (10.5)
Aboriginal or Torres Strait Islanders	8 (2.0)	3 (0.8)
Polynesian	8 (2.0)	8 (2.1)
Maori	10 (2.5)	10 (2.6)
Other	43 (10.9)	38 (9.8)
BMI category		
Underweight	16 (4.0)	18 (4.6)
Normal	156 (39.2)	147 (37.8)
Overweight	94 (23.6)	93 (23.9)
Obese	100 (25.1)	93 (23.9)
Unknown	32 (8.0)	38 (9.8)
Progesterone use < 16 weeks’ gestation	14 (3.5)	12 (3.1)
Gestational age at randomization (weeks)^#^	20.6 (19.3, 22.1)	20.4 (19.3, 22.0)
Main reason for previous preterm birth		
Preterm labour	256 (64.3)	235 (60.4)
PPROM	127 (31.9)	140 (36.0)
Other	15 (3.8)	14 (3.6)
Gestational age of previous preterm birth		
<28 weeks’	124 (31.2)	118 (30.3)
28 to <34 weeks’	133 (33.4)	128 (32.9)
≥34 weeks’	141 (35.4)	143 (36.8)
Current pregnancy		
Singleton	390 (98.0)	385 (99.0)
Twins	8 (2.0)	4 (1.0)
Study treatment used	381 (95.7)	359 (92.3)
Study treatment taken (days)^#^	51.0 (28.0, 69.0)	52.0 (27.0, 76.0)

Values are number (percentage), unless otherwise indicated. BMI, body mass index; CI, confidence interval; PPROM, preterm prelabour rupture of membranes.

* Values are means (standard deviation).

^‡^ Ethnicity as reported by the participant.

^#^ Values are medians (interquartile range).

### Primary infant outcomes

#### Risk of respiratory distress syndrome and severity of respiratory disease

The risk of respiratory distress syndrome was similar in both study groups, 10.5% (42/402) in the progesterone group and 10.6% (41/388) in the placebo group (adjusted RR 0.98, 95% CI 0.64–1.49, *p* = 0.912), as was the severity of any neonatal respiratory disease (adjusted treatment effect 1.02 [95% CI 0.69–1.53, *p* = 0.905]) (Table 2). Unadjusted analyses showed similar findings to the analyses adjusted for gestational age at randomisation, gestation of previous preterm birth, and reason for previous preterm birth (Table 2).

**Table 2:** Primary and secondary neonatal outcomes by study group.

Outcome	Progesterone *n* = 406	Placebo *n* = 393		Unadjusted Treatment Effect for Progesterone versus Placebo (95% CI)	Unadjusted *p*-Value	Adjusted Treatment Effect for Progesterone versus Placebo (95% CI)	Adjusted *p*-Value^#^
*Primary Outcomes*							
Neonatal RDS	42/402 (10.5)	41/388 (10.6)		0.99 (0.65–1.51)	0.958	0.98 (0.64–1.49)	0.912
Severity of respiratory disease*				1.03 (0.69–1.53)	0.883	1.02 (0.69–1.53)	0.905
Nil	338/402 (84.1)	327/388 (84.3)					
Mild	24/402 (6.0)	28/388 (7.2)					
Moderate	32/402 (8.0)	26/388 (6.7)					
Severe	8/402 (2.0)	7/388 (1.8)					
*Secondary Outcomes*							
*Other Respiratory Measures*							
Oxygen therapy	43/402 (10.7)	45/388 (11.6)		0.92 (0.61–1.38)	0.696	0.92 (0.62–1.37)	0.670
Duration of oxygen therapy**	4.96 (38.0)	5.43 (45.7)		0.91 (0.29–2.84)	0.875	0.85 (0.32–2.29)	0.751
Highest FiO_2_ at <12 hours of birth^‡^	29.6 (13.9)	27.9 (13.0)		1.68 (−3.03 to 6.38)	0.485	1.37 (−2.97 to 5.71)	0.536
Mechanical ventilation	26/402 (6.5)	23/388 (5.9)		1.09 (0.62–1.92)	0.763	1.08 (0.62–1.89)	0.788
Duration of mechanical ventilation (days)**	0.50 (3.0)	0.70 (4.8)		0.72 (0.29–1.79)	0.477	0.51 (0.21–1.24)	0.137
Surfactant used	26/402 (6.5)	25/388 (6.4)		1.00 (0.58–1.74)	0.989	0.99 (0.58–1.72)	0.984
Nitric oxide for respiratory support	0/402 (0.0)	3/388 (0.8)		N/A	0.118^	N/A	N/A
Air leak syndrome	2/402 (0.5)	1/388 (0.3)		N/A	1.000^	N/A	N/A
Chronic lung disease	10/402 (2.5)	7/388 (1.8)		1.38 (0.49–3.87)	0.542	N/A	N/A
*Adverse Infant Outcomes*							
Serious infant outcome (composite)	155/406 (38.2)	152/393 (38.7)		0.99 (0.82–1.18)	0.887	0.98 (0.82–1.17)	0.798
Preterm birth at <37 weeks’ gestation	148/406 (36.5)	146/393 (37.2)		0.98 (0.81–1.18)	0.842	0.97 (0.81–1.17)	0.765
Perinatal mortality	5/406 (1.2)	7/393 (1.8)		0.69 (0.22–2.16)	0.526	N/A	N/A
Stillbirth	4/406 (1.0)	5/393 (1.3)		N/A	0.749^	N/A	N/A
Infant death	1/406 (0.3)	2/393 (0.5)		N/A	0.619^	N/A	N/A
Born by caesarean section	126/406 (31.0)	105/393 (26.7)		1.16 (0.93–1.45)	0.187	1.17 (0.94–1.46)	0.160
Apgar score < 4 at 5 minutes	6/406 (1.5)	6/393 (1.5)		0.97 (0.31–2.98)	0.955	N/A	N/A
Birth weight (g) ^‡^	2870.2 (849.2)	2926.5 (794.2)		−56.3 (−174.9 to 62.4)	0.353	−53.6 (−171.7 to 64.4)	0.373
Birth weight z-score^‡^	0.13 (1.1)	0.20 (1.0)		−0.07 (−0.22 to 0.07)	0.333	−0.07 (−0.21 to 0.08)	0.356
Birth weight < 3rd centile	8/402 (2.0)	7/388 (1.8)		1.10 (0.38–3.19)	0.856	N/A	N/A
Any IVH	9/402 (2.2)	9/388 (2.3)		0.97 (0.39–2.41)	0.939	N/A	N/A
Grade 3/4 IVH	1/402 (0.3)	1/388 (0.3)		N/A	1.000^	N/A	N/A
Periventricular leucomalacia	0/402 (0.0)	1/388 (0.3)		N/A	0.491^	N/A	N/A
Inotropic support for PDA	11/402 (2.7)	9/388 (2.3)		1.18 (0.47–2.96)	0.725	N/A	N/A
Necrotising enterocolitis	2/402 (0.5)	2/388 (0.5)		N/A	1.000^	N/A	N/A
Proven early neonatal sepsis	0/402 (0.0)	2/388 (0.5)		N/A	0.241^	N/A	N/A
Retinopathy of prematurity	12/401 (3.0)	9/386 (2.3)		1.28 (0.51–3.26)	0.600	N/A	N/A
Admission to NICU	68/402 (16.9	71/388 (18.3)		0.92 (0.68–1.27)	0.624	0.92 (0.67–1.25)	0.591
Infant postnatal length of stay (days)**	11.93 (21.1)	11.24 (21.6)		1.06 (0.80–1.41)	0.677	1.06 (0.79–1.41)	0.717

Denominators are 406 in the progesterone group and 393 in the placebo group for outcomes that include all infants alive at the time of randomisation and 402 and 388, respectively, for outcomes relating to only live-born infants (where the 4 stillbirths in the progesterone group and 5 stillbirths in the placebo group are not included). Values are number (percentage), and treatment effects are relative risks unless otherwise indicated. Abbreviations: CI, confidence interval; FIO_2_, fractional inspired oxygen; GA, gestational age; IVH, intraventricular haemorrhage; N/A, not available; NICU, neonatal intensive care unit; PDA, patent ductus arteriosus; RDS, respiratory distress syndrome.

^#^ Adjusted for GA at randomization, GA of previous preterm birth, and reason for previous preterm birth.

* Values are number (percentage), and treatment effects are odds ratios of higher severity.

** Values are mean (standard deviation), and treatment effects are ratios of means.

^‡^ Values are mean (standard deviation), and treatment effects are differences in means.

^ *p*-Value from Fisher's exact test.

### Secondary outcomes for the infant

#### Other respiratory measures

In keeping with these findings, there were no differences between the study groups for any of the secondary respiratory outcomes that included need for and duration of oxygen therapy, maximum appropriate FIO_2_ values within 12 hours of birth, use and duration of mechanical ventilation, use of surfactant, use of nitric oxide, air leak syndrome, and chronic lung disease (Table 2).

#### Adverse infant outcomes

Overall, the risk of any serious adverse outcome for the infant was similar between the study groups (155/406 [38.2%] in the progesterone group and 152/393 [38.7%] in the placebo group, adjusted RR 0.98, 95% CI 0.82–1.17, *p* = 0.798) (Table 2). There were 12 (1.5%) infant deaths before hospital discharge: 4 stillbirths and 1 death of a live-born infant in the progesterone group and 5 stillbirths and 2 deaths of live-born infants in the placebo group—not a significant difference (Table 2). The proportion of infants born before 37 weeks’ gestation was similar in both study groups (148/406 [36.5%] in the progesterone group and 146/393 [37.2%] in the placebo group, adjusted RR 0.97, 95% CI 0.81–1.17, *p* = 0.765). A similar proportion of infants were born by caesarean section in both study groups. No differences were evident between the study groups for any of the other individual adverse infant outcomes that included low Apgar score, small for gestational age at birth, intraventricular haemorrhage, periventricular leucomalacia, patent ductus arteriosus requiring treatment, necrotising enterocolitis, proven early neonatal sepsis, retinopathy of prematurity, and need for admission to the neonatal intensive care unit and duration of the infant’s postnatal stay (Table 2).

### Secondary outcomes for the women

#### Significant health outcomes

There were no differences between study groups in the proportion of women experiencing 1 or more significant health outcomes overall (180/398 [45.2%] in the progesterone group and 174/389 [44.7%] in the placebo group, adjusted RR 1.00, 95% CI 0.86–1.17, *p* = 0.994) or in the individual health outcomes, particularly those related to preterm birth, including use of tocolytic therapy and antenatal corticosteroids prior to the birth, antepartum haemorrhage, preeclampsia, risk of rupture of the membranes preterm or at or near term, chorioamnionitis requiring antibiotics, and postpartum haemorrhage (Table 3). There were no maternal deaths. Antibiotic use after birth was similar between the study groups, as was the need for antenatal admission and the length of any antenatal or postnatal hospital stay (Table 3).

**Table 3:** Secondary maternal outcomes by study group, including those related to preterm birth, psychological health, side effects of study treatment, and compliance.

Outcome	Progesterone (*n* = 398)	Placebo (*n* = 389)	Unadjusted Treatment Effect for Progesterone versus Placebo (95% CI)	Unadjusted *p*-Value	Adjusted Treatment Effect for Progesterone versus Placebo (95% CI)	Adjusted *p*-Value^#^
*Significant Health Outcomes*						
Serious maternal outcome	180/398 (45.2)	174/389 (44.7)	1.01 (0.87–1.18)	0.889	1.00 (0.86–1.17)	0.994
Tocolytic therapy	71/398 (17.8)	77/389 (19.8)	0.90 (0.67–1.21)	0.483	0.90 (0.68–1.20)	0.478
Antenatal corticosteroid treatment	147/398 (36.9)	143/389 (36.8)	1.00 (0.84–1.21)	0.960	1.01 (0.84–1.21)	0.926
Antepartum haemorrhage	17/398 (4.3)	20/389 (5.1)	0.83 (0.44–1.56)	0.565	0.83 (0.44–1.56)	0.561
Pre-eclampsia	12/398 (3.0)	8/389 (2.1)	1.47 (0.61–3.55)	0.396	N/A	N/A
Preterm prelabour rupture of membranes	51/398 (12.8)	44/389 (11.3)	1.13 (0.78–1.65)	0.518	1.14 (0.78–1.66)	0.500
Term prelabour rupture of membranes	24/398 (6.0)	25/389 (6.4)	0.94 (0.55–1.61)	0.818	0.92 (0.54–1.58)	0.772
Chorioamnionitis requiring antibiotics	19/398 (4.8)	13/389 (3.3)	1.43 (0.72–2.85)	0.312	1.49 (0.75–2.98)	0.253
Postpartum haemorrhage ≥ 500 ml	88/398 (22.1)	91/389 (23.4)	0.95 (0.73–1.22)	0.668	0.95 (0.73–1.22)	0.682
Postnatal antibiotic use	43/398 (10.8)	33/389 (8.5)	1.27 (0.83–1.96)	0.272	1.29 (0.84–1.98)	0.247
Antenatal hospitalization	191/398 (48.0)	186/389 (47.8)	1.00 (0.87–1.16)	0.961	1.01 (0.88–1.16)	0.908
Length of antenatal hospitalization (days)**	3.82 (10.6)	3.46 (7.8)	1.10 (0.82–1.49)	0.523	1.02 (0.76–1.37)	0.919
Length of postnatal hospitalization (days)**	2.72 (2.1)	2.60 (1.9)	1.05 (0.95–1.16)	0.346	1.05 (0.95–1.15)	0.351
*Psychological Health at 34 Weeks’* ^##^						
Quality of life (SF-36) domains						
Physical functioning*	54.04 (26.3)	55.81 (27.1)	−1.77 (−6.30 to 2.77)	0.445	−1.30 (−5.77 to 3.18)	0.570
Physical role*	38.35 (40.7)	44.91 (41.5)	−6.56 (−13.54 to 0.42)	0.066	−6.62 (−13.55 to 0.32)	0.061
Bodily pain*	59.60 (22.7)	59.90 (24.8)	−0.30 (−4.33 to 3.73)	0.884	−0.52 (−4.53 to 3.49)	0.799
General health*	76.61 (17.8)	75.08 (17.8)	1.53 (−1.50 to 4.55)	0.323	1.47 (−1.56 to 4.50)	0.342
Vitality*	49.44 (20.0)	50.45 (20.5)	−1.02 (−4.46 to 2.42)	0.562	−1.06 (−4.51 to 2.38)	0.546
Social functioning*	69.55 (27.0)	73.35 (25.7)	−3.80 (−8.28 to 0.67)	0.096	−3.76 (−8.22 to 0.69)	0.098
Emotional role*	82.21 (32.3)	82.52 (33.6)	−0.31 (−5.90 to 5.28)	0.913	−0.21 (−5.78 to 5.36)	0.941
Mental health*	76.92 (17.9)	77.24 (16.2)	−0.33 (−3.23 to 2.58)	0.827	−0.27 (−3.16 to 2.61)	0.853
Overall physical component*	37.43 (9.8)	38.32 (10.6)	−0.90 (−2.63 to 0.84)	0.312	−0.85 (−2.58 to 0.87)	0.333
Overall mental component*	51.95 (10.4)	52.23 (9.4)	−0.28 (−1.97 to 1.40)	0.743	−0.30 (−1.98 to 1.38)	0.724
Anxiety (STAI score)*	10.91 (3.9)	11.02 (3.7)	−0.11 (−0.75 to 0.53)	0.739	−0.10 (−0.74 to 0.54)	0.763
Depression (EPDS score > 12)	25/266 (9.4)	24/266 (9.0)	1.04 (0.61–1.78)	0.881	1.05 (0.62–1.78)	0.868
*Side Effects of Therapy and Compliance*						
Women reporting side effects	134/394 (34.0)	118/382 (30.9)	1.10 (0.90–1.35)	0.354	1.11 (0.90–1.36)	0.322
Side effects reported						
Headache	39/394 (9.9)	35/382 (9.2)	1.08 (0.70–1.67)	0.727	1.07 (0.69–1.65)	0.769
Nausea	33/394 (8.4)	24/382 (6.3)	1.33 (0.80–2.21)	0.266	1.33 (0.80–2.21)	0.269
Pain or discomfort	29/394 (7.4)	29/382 (7.6)	0.97 (0.59–1.59)	0.903	0.96 (0.59–1.56)	0.861
Breast tenderness	12/394 (3.1)	16/382 (4.2)	0.73 (0.35–1.52)	0.396	0.72 (0.34–1.49)	0.372
Coughing	10/394 (2.5)	5/382 (1.3)	1.94 (0.67–5.62)	0.223	N/A	N/A
Other	66/394 (16.8)	58/382 (15.2)	1.10 (0.80–1.52)	0.552	1.13 (0.82–1.55)	0.469
Treatment stopped because of side effects	39/394 (9.9)	28/382 (7.3)	1.35 (0.85–2.15)	0.205	1.35 (0.85–2.15)	0.204
Noncompliant with treatment (did not start treatment or forgot to use ≥ 3 times a week)	33/394 (8.4)	35/380 (9.2)	0.91 (0.58–1.43)	0.682	0.93 (0.59–1.46)	0.743
Did not start treatment or stopped before 34 weeks’ gestation	131/381 (34.4)	113/360 (31.4)	1.10 (0.89–1.35)	0.3867	1.08 (0.88–1.33)	0.454

Values are number (%), and treatment effects are relative risks unless otherwise indicated. Term prelabour rupture of membranes was adjusted for gestational age at randomisation and gestational age of previous preterm birth only. Experienced side effects: headache and nausea were adjusted for gestational age at randomisation and gestational age of previous preterm birth only. Abbreviations: CI, confidence interval; EPDS, Edinburgh Postnatal Depression Scale [25]; SF-36, 36-Item Short Form Health Survey [23]; STAI, State-Trait Anxiety Inventory [24].

# Adjusted for gestational age at randomisation, gestational age of previous preterm birth, and reason for previous preterm birth.

## The denominators are 266 for progesterone and 265 for placebo.

* Values are mean (standard deviation), and treatment effects are differences in means.

** Values are mean (standard deviation), and treatment effects are ratios of means.

#### Psychological health

All measures on the 36-Item Short Form Health Survey (SF-36), including the overall physical and mental components, were similar in both study groups. No differences were seen in the proportion of women with a score on the Edinburgh Postnatal Depression Scale (EPDS) that was suggestive of depression (9.4% in the progesterone group and 9.0% in the placebo group), and the level of anxiety was similar in the 2 study groups (Table 3).

#### Side effects of study treatment and compliance

The proportion of women reporting any side effects of the treatment at 34 weeks’ gestation was similar between the study groups (134/394 [34.0%] in the progesterone group versus 118/382 [30.9%] in the placebo group), as was the proportion of women who stopped therapy because of side effects (39/394 [9.9%] in the progesterone group versus 28/382 [7.3%] in the placebo group) (Table 3). A similar proportion of women in both study groups either did not start the medication or forgot to use it 3 or more times a week, our measure of compliance (33/394 [8.4%] in the progesterone group and 35/380 [9.2%] in the placebo group) (Table 3). A similar proportion of women in both study groups used the study treatment up to 34 weeks’ gestation and remained undelivered (250/381 [65.6%] in the progesterone group versus 247/360 [68.6%] in the placebo group).

## Discussion

### Main findings

The PROGRESS Trial showed that in women with a history of previous spontaneous preterm birth, the use of 100-mg vaginal progesterone pessaries daily from 20 weeks’ gestation until 34 weeks’ gestation had no effect on the risk of the baby developing respiratory distress syndrome or on reducing the severity of any neonatal respiratory disease compared with placebo pessaries. In keeping with these findings, no benefits were seen relating to other respiratory outcomes or other neonatal morbidities.

For women, the risk of having a preterm birth was not reduced with the use of progesterone, and the need for interventions related to preterm birth such as tocolysis and antenatal corticosteroids; the need for antenatal hospital admission; and, if admitted, the length of hospital stay were also not reduced. Over 36% of the women in both study groups in the PROGRESS Trial were given antenatal corticosteroids, appropriate for the 36% rate of preterm birth seen in our high-risk population. Although progesterone can suppress proinflammatory cytokines [27], there was no evidence that progesterone exerted an anti-inflammatory effect on infective outcomes for the mother or the baby such as chorioamnionitis requiring the use of antibiotics, need for antibiotic use after birth, or the infant having proven early sepsis. Maternal psychological health status was similar in both study groups, including vulnerability to depression. This is reassuring given the concern that progesterone could aggravate depression.

### Generalisability and comparison with other studies

We found no effect of vaginal progesterone on the risk of preterm birth for women with a previous preterm birth, similar to the findings from the O’Brien Trial [16] and the recently published OPPTIMUM Trial [28] but in contrast to other published reports [15, 29, 30, 31,32].

### Strengths of the PROGRESS Trial

The clear entry criteria for the PROGRESS Trial were specifically set to easily identify women at high risk of a recurrence of preterm birth based on their previous history and to assess the effects of vaginal progesterone on this population. Inclusion criteria for our study were based on a previous history of preterm birth—a strong predictor for subsequent preterm birth—and not dependent on assessment of cervical length. Women identified and recruited with a history of preterm birth in their preceding pregnancy were at high risk of recurrence, with 36% giving birth before 37 weeks’ gestation, although there was no difference in gestational age at birth or in the proportion born preterm between the study groups. The trial was masked for participants and investigators with a placebo, and the primary outcome of respiratory distress syndrome was reported for all babies.

### Potential limitations of current trial

It is possible that the dose of 100 mg progesterone used may have been too low. However, the Da Fonseca Trial [15] used the same 100-mg dose of vaginal progesterone and included women at high risk for preterm birth, defined by at least 1 previous spontaneous preterm birth, prophylactic cervical cerclage, or uterine malformation, but reported a lower rate of preterm birth compared with placebo (13.8% versus 28.5%), as have other trials [29,31]. Of note, a larger daily dose of 200 mg as used in the OPPTIMUM Trial was not found to reduce the risk of preterm birth or improve neonatal or child health at 2 years of age [28].

Our pretrial sample size estimate, based on the reported effect of treatment with progesterone compared with placebo on neonatal respiratory distress syndrome [21], would provide 80% to detect a difference at the 5% significance level. Whilst the reduction in sample size recommended at the masked interim review of data may have reduced power to detect differences, the final trial results do provide reliable study estimates with CIs. To show differences between treatment groups based on these study estimates at the 5% significance level and with 80% power would require a sample size of over 2,966,780 women.

Of eligible women invited to participate in the PROGRESS Trial, only 41% chose to do so, not too dissimilar to the 52% consent rate in the OPPTIMUM Trial [28]. Whether greater involvement of consumers in research proposals and promotion of trials open for recruitment within the community can increase participation in preterm birth research in priority areas, already identified by consumers of care and healthcare practitioners, needs to be established [1,33].

In any intervention study, compliance is crucial to ascertain true effect. Few other studies to date have reported on measures of compliance. In our study, most women started the allocated study treatment, and the median days of use was around 51 days. Nevertheless, a proportion of women in both study groups, almost 9%, either did not start the medication or forgot to use it 3 or more times a week, which was our measure of compliance. Within the OPPTIMUM Trial, compliance—defined slightly differently as 80% or more use of study treatment—was 69% [28]. This is similar to the proportion of women in the PROGRESS Trial who were still taking their study treatment and remained undelivered up to 34 weeks’ gestation (65.2% for women in the progesterone group and 68.6% in the placebo group).

Almost a third of the women reported side effects of treatment with the vaginal pessaries, the most frequent reasons given being headache, nausea, and pain or discomfort, although there were no differences in the proportion of women reporting side effects or the side effects reported by study group. For over 8% of women, these side effects were sufficient for them to stop their study treatment. Cessation of therapy because of side effects has not been well reported in earlier studies.

### Clinical relevance

There are ongoing differences in clinical practice recommendations as to whether to recommend use of progesterone or not [17,18,19,20]. The critical issues are whether there are particular subgroups of women who may benefit from use of vaginal progesterone by virtue of their previous obstetric history (such as a history of preterm birth or factors in their current pregnancy, such as shortening of the cervix) and what is the optimal dose and treatment regimen to use (including the gestational age to start treatment, the length of time to use treatment, and the optimal mode of administration: vaginal or intramuscular preparation). There have been calls for an individual participant data meta-analysis (IPD-MA) of the trials already conducted [28, 34] that we strongly endorse. An IPD-MA can assess different participant- and treatment-level characteristics, which is not possible using an aggregate meta-analysis, and thus provide cumulated evidence on these critical issues identified that can be used by women and their families, clinicians, and policy makers as well as identify future research priorities.

### Conclusions

#### Recommendations for clinical practice

Our results do not support the use of vaginal progesterone pessaries in women with a history of a previous spontaneous preterm birth to reduce the risk of respiratory distress syndrome or other neonatal or maternal morbidity. IPD-MA of the relevant trials may identify specific women for whom vaginal progesterone may be of benefit. The search for alternative strategies for the prevention of preterm birth and its sequelae must continue.

## Supporting information

S1 Text Consolidated Standards of Reporting Trials (CONSORT) statement. (DOCX)

S2 Text Trial protocol for the PROGRESS study. (DOCX)

## Abbreviations

BMI: body mass index
CI: confidence interval
CONSORT: Consolidated Standards of Reporting Trials
EPDS: Edinburgh Postnatal Depression Scale
FIO_2_: fractional inspired oxygen
GA: gestational age
IPD-MA: individual participant data meta-analysis
IQR: interquartile range
IVH: intraventricular haemorrhage
MAP: mean airway pressure
NICU: neonatal intensive care unit
PDA: patent ductus arteriosus
PPROM: preterm prelabour rupture of the membranes
RDS: respiratory distress syndrome
RR: relative risk
SF-36: 36-Item Short Form Health Survey
STAI: State-Trait Anxiety Inventory
