# Supplementary material for: Vaginal progesterone pessaries for pregnant women with a previous preterm birth to prevent neonatal respiratory distress syndrome (the PROGRESS Study): A multicentre, randomised, placebo-controlled trial
Source: PLoS Med. 2017 Sep 26;14(9):e1002390. doi: 10.1371/journal.pmed.1002390 (PMC5614421; doi:10.1371/journal.pmed.1002390)
Supplement: S2 Text — (DOCX) [file pmed.1002390.s002.docx]

# PROTOCOL

# Progesterone after previous preterm birth for the prevention of

# neonatal Respiratory distress Syndrome

COORDINATING CENTRE:

Perinatal Clinical Trials Unit

Department of Obstetrics and Gynaecology

The University of Adelaide

Women’s and Children’s Hospital

King William Road

North Adelaide

South Australia

SA 5006

Tel No. +61 8 8161 7617

Fax. +61 8 8161 7652

Email: [caroline.crowther@adelaide.edu.au](mailto:caroline.crowther@adelaide.edu.au)

Version 22/10/06

Contents

page

1. Title 3

2. Investigators 3

3. Aims 3

4. Hypotheses 3

5. Background 4

6. Research Plan

- Study Design 9

- Inclusion Criteria 9

- Exclusion Criteria 9

- Trial Entry 9

- Study Medication and Treatment Schedules 10

- Study Endpoints 10

- Sample Size 11

- Committee Structure 12

- Analysis and Reporting of Results 12

- Confidentiality & Data Security 12

- Outcome and Significance 12

7. References 13

**TITLE: Progesterone after previous preterm birth for prevention of neonatal Respiratory distress Syndrome**

**INVESTIGATORS**

Professor Caroline Crowther

Dr Jodie Dodd

Dr Andrew McPhee

Ms Vicki Flenady

Professor Jeffrey Robinson

**AIMS OF THE TRIAL**

Respiratory distress syndrome, as a consequence of preterm birth, is the major cause of early neonatal mortality and morbidity. Amongst survivors, there remains considerable risk of chronic lung disease and long-term neurological disability. Progesterone is involved in maintaining uterine quiescence, and its withdrawal, either actual or functional, is an antecedent to the onset of labour. Although recent reports of progesterone supplementation for women at risk of preterm birth show promise, there is currently insufficient data on clinically important outcomes to enable informed decision-making.

### SPECIFIC AIMS

The aims of this randomised, double blind, placebo controlled trial are to assess whether the use of vaginal progesterone pessaries in women with a history of previous spontaneous preterm birth will reduce the risk and severity of respiratory distress syndrome, so improving their infant’s health, without increasing maternal risks.

**TRIAL HYPOTHESES**

**The primary hypothesis** of this randomised trial is that the administration of progesterone to women with a history of previous spontaneous preterm birth will

• **reduce the risk of neonatal respiratory distress syndrome**.

**The secondary hypotheses** of this randomised trial are that the administration of progesterone to women with a history of previous spontaneous preterm birth will

• **reduce the risk of morbidity from neonatal lung disease**;

• **reduce the risk of morbidity from other adverse outcomes for the infant;**

• **reduce the risk of maternal morbidity without adverse effects of therapy**.

**BACKGROUND**

***Respiratory distress syndrome: the burden of disease***

Respiratory distress syndrome, as a consequence of immature lung development, is a significant consequence of preterm birth and the major cause of early neonatal mortality and morbidity (Kramer 2000). Infants born very preterm often require respiratory support, with significant numbers requiring mechanical ventilation (Donoghue 2002). Of infants born very preterm who survive, 20% remain dependent on oxygen therapy 28 days after birth, and 25% are diagnosed with chronic lung disease (Donoghue 2002). There is a recognised increased risk of hospitalisation in the first year of life (Elder 1999), and risk of subsequent long-term handicap and cerebral palsy (Stanley 1992).

The incidence of preterm birth (birth less than 37 weeks gestation) in Australia in 2002 was 7.0% (Laws 2002) (Australia's mothers and babies 2000). This relatively small proportion of all births accounted for over 70% of the total perinatal mortality (Australia's mothers and babies 2000). Each year over 20,000 women in Australia give birth to a preterm infant at less than 37 weeks gestation and for 50% of these women, birth is due to spontaneous preterm labour or preterm ruptured membranes (Hewitt 1988, McLaughlin 2002).

The immediate and long-term monetary costs of preterm birth and its associated neonatal intensive care unit admission are high. In the United States in 1990, the weekly cost of approximately $US10,000 per preterm baby gave an estimated annual total cost in excess of $5 billion (Morrison 1990). The mean cost to provide care for a premature infant from admission to discharge from the newborn intensive care unit was between $20,000 and $100,000 per infant, rising to $140,000 for those weighing less than 1000 grams. Infants with severe disability have long-term care costs estimated to be more than $100,000 and lifetime custodial care as much as $450,000 (Morrison 1990). The emotional and personal costs to families and individuals who are born preterm are considerable.

***The likelihood of recurrence of preterm birth***

Women who have given birth preterm in one pregnancy are more likely to give birth preterm in a subsequent pregnancy (Bakketeig 1979, Adams 2000, Bloom 2001). Petrini and colleagues have estimated the rate of recurrent preterm birth to be 22.5% (Petrini 2005), a 2.5 times increased risk, compared with women with no previous spontaneous preterm birth (Mercer 1999). Population based cohort data suggests that for women who give birth between 20 and 31 weeks gestation in one pregnancy, 29.3% will give birth preterm before 37 weeks in a subsequent pregnancy. The preterm birth occurs at a similar gestation for 8.2% of these women (Adams 2000).

***Respiratory distress syndrome: How can we reduce the burden of disease?***

Reducing the risk of respiratory distress syndrome requires preventing preterm birth, or improving lung maturation in infants born preterm. Prevention of preterm birth remains elusive. In high income countries the incidence of preterm birth is estimated at 6% to 10% of all births and has remained unchanged over the last four decades despite intensive antenatal care programs aimed at high-risk groups, the widespread use of pharmacological agents to inhibit preterm birth (tocolytics) and a series of other preventive and therapeutic interventions. Identification and treatment of women with vaginal infection holds new promise (Kiss 2004, McDonald 2005).

Antenatal corticosteroids administered to women at risk of preterm birth remain the ‘gold standard’ prenatal intervention to reduce the risk of neonatal respiratory distress syndrome (Crowley 2005), although even with appropriate treatments the risk of respiratory distress syndrome remains considerable (ACTOBAT 1995). The risk of respiratory distress syndrome in an Australian population, after exposure to a single dose of prenatal corticosteroids, is 27% with 12.4% of infants having severe disease (ACTOBAT 1995).

The use of progesterone as an agent to prevent preterm birth dates back to the early 1960’s (LeVine 1964)). There has been renewed interest in this agent as a means of reducing the sequelae of preterm birth and in particular neonatal respiratory distress syndrome, with the recent publication of two clinical trials (da Fonseca 2003, Meis 2003). What then is the role of progesterone in the initiation of labour?

***The role of progesterone in the initiation of labour***

The exact mechanism of the onset of parturition in humans is a complex interaction of many different hormonal pathways (Challis 2000, Challis 2002, Gibb 2002, Lopez 2003). Progesterone is essential for the maintenance of pregnancy (Pepe 1995, Pieber 2001, Astle 2003), having an important role in uterine quiescence (Grazzini 1998, deZiegler 2001). Progesterone contributes to uterine quiescence through suppression of the calcium-calmodulin-myosin light chain kinase system, reducing calcium flux and altering the resting potential of smooth muscle (Lopez 2003, Pepe 1995).

Debate remains about the relationship between progesterone withdrawal (Csapo 1956) and the onset of labour (Mesiano 2004). In humans, the progesterone receptor (PR) has two major subtypes PR-A and PR-B. PR-A represses the actions of progesterone mediated by PR-B (Mesiano 2004). An increase in the myometrial PR-A to PR-B expression ratio occurs at the onset of labour at term, resulting in an increase in myometrial PR-A, and in effect a functional withdrawal of progesterone (Mesiano 2004, Smith 2002), with increasing sensitivity to contractile stimuli (Pieber 2001, Astle 2003, Haluska 2002, Condon 2003).

Before birth, coordinated uterine activity is associated with connective tissue changes resulting in cervical ripening and dilatation (Lopez 2003). In many animals this is associated with a decrease in progesterone concentrations (Challis 2000, Lopez 2003, Astle 2003, Jenkin 1985). This has not been shown to occur in women before term or preterm birth, with no apparently detectable changes to circulating steroid hormone levels evident (Lopez 2003, Pieber 2001, Astle 2003, Block 1984, Smit 1984).

Pharmacological withdrawal of progesterone with 3-beta-hydroxysteroid dehydrogenase inhibitors or mifepristone (RU486) is associated with the onset of labour, and has been used clinically in facilitating the termination of pregnancy (Rodger 1987, el-Refaey 1992). In contrast, progesterone pessaries have been used to maintain pregnancy in women undergoing assisted reproductive techniques until placental production is sufficient (Nyboe-Andersen 2002).

Progesterone is known to have anti-inflammatory properties, raising a possible link between inflammatory process, alterations in progesterone receptor expression and the onset of preterm labour and may have a role in potentiating the action of tocolytics on the myometrium, with natural progesterone shown to increase the relaxant effect of ritodrine (Smith 2004).

***Pharmacokinetics of progesterone by route of administration***

The currently available information about pharmacokinetics relates to the use of progesterone in assisted reproduction (Abate 1997, Levy 1999, Tavaniotou 2000, Penzias 2002), in menopausal (Cicinelli 1993) and post-menopausal women (Levy 1999, Mircioio 1999), and in women with endometrial carcinoma (Onsrud 1985). These studies indicate blood progesterone concentrations following vaginal administration to be lower than after intramuscular administration (Tavaniotou 2000, Penzias 2002), although there is little data available to inform the optimal route of administration in women in later pregnancy.

For 100mg vaginal progesterone pessaries the peak blood concentrations are obtained 3-8 hours after vaginal administration, due to avoidance of first pass metabolism in the liver. In blood, progesterone is 96-99% protein bound, mainly to albumin.

While there may be advantages in the use of intramuscular progesterone in terms of increased blood concentrations, such preparations are not available for prescription within Australia. Natural progesterone has advantages over synthetic forms in cost, safety profile and availability.

***Safety of progesterone***

Natural progesterone has been used in pregnancy without demonstrated effect on fetal development or on the risk of congenital anomalies (Schardein 1980, Raman 1995), although continuous exposure during pregnancy to steroid hormones remains a concern. Information from animal studies suggests that progesterone influences fetal behaviour in sheep (Crossley 1997), with increased concentrations suppressing activity and arousal states (Nicol 1997, Nicol 1999). Clearly further evaluation of potential harmful effects to the fetus is required.

Maternal side effects related to progesterone therapy include headache, nausea, breast tenderness, and coughing.

**“Is there clinical evidence to suggest a role for progesterone in reducing the risk of respiratory distress syndrome as a sequelae of preterm birth?”**

***Appraising the evidence: The Cochrane Review***

The Cochrane systematic review (Dodd 2006) to assess the use of progesterone in pregnancy identified seven randomised controlled trials, comparing intramuscular 17 alphahydroxyprogesterone caproate with placebo (LeVine 1964, Meis 2003, Papiernik, Johnson 1975, Hartikainen 1980, Hauth 1983, Yemini 1985). Two trials were excluded as they adopted a quasi-randomised method of treatment allocation (LeVine 1964, Yemini 1985).

***Methodology of the Review:***

**Types of Studies:** Published RCTs

**Participants:** Pregnant women

**Interventions:** Progesterone therapy

**Databases:** Cochrane Controlled Trials Register (CENTRAL) and MEDLINE (last searched January 2005)

**Search Terms:** progesterone, pregnancy, preterm birth, preterm labour, and randomised controlled trial

## Intramuscular progesterone versus placebo:

We have combined the data from the five randomised trials that have compared intramuscular progesterone with placebo in a meta-analysis.

Women receiving progesterone had a statistically significant lower risk of preterm birth and in keeping with this, fewer infants with birth weight less than 2.5kg when compared with those women administered placebo. There is insufficient evidence to assess the effects of progesterone therapy on perinatal death or respiratory distress syndrome, with a single trial only (Meis 2003) reporting any neonatal morbidity data.

However, **intra-muscular 17 alpha-hydroxyprogesterone caproate is not available for general prescription within Australia**, or within the United States of America or the United Kingdom.

***Vaginal progesterone pessaries versus placebo:***

**Only one trial was identified comparing vaginal progesterone pessaries with placebo (da Fonseca 2003).**

Da Fonsesca and colleagues (da Fonseca 2003) randomised 157 asymptomatic women in Brazil with a singleton pregnancy considered to be at “high risk” of preterm birth (history of previous preterm birth, the presence of a cervical suture, or uterine malformation), to receive either 100mg progesterone or placebo suppositories administered nightly between 24 and 34 weeks gestation (da Fonseca 2003). Fewer women in the progesterone group gave birth before 37 weeks gestation (10/72 (13.9%) progesterone versus 20/70 (28.6%) placebo; relative risk 0.49; 95% Confidence Intervals 0.25-0.96; p=0.03). **No infant outcomes were reported**.

***Limitations of the current evidence on the use of vaginal progesterone***

• **No reporting of any infant outcomes**

• **No reporting of any maternal outcomes**

• **Lack of information about potential harmful effects for the fetus from prolonged progesterone exposure**

• **Small sample size of single trial, and therefore underpowered to detect differences in relevant clinical outcomes**

• **No trials have been conducted in an Australasian setting**

• **Therefore, there is insufficient information to make recommendations about the benefits and harms of progesterone therapy.**

***In Summary***

**We propose that a randomised, double blind, placebo controlled trial to assess the use of vaginal progesterone therapy for women with a history of previous preterm birth, to reduce respiratory distress syndrome and so improve infants’ health is needed and timely for the following reasons:**

1. Respiratory distress disease remains the cause of neonatal mortality and morbidity.

2. It is estimated that each year in Australia there are over 20,000 pregnant women who have had a previous spontaneous preterm birth before 37 weeks gestation.

3. Progesterone is involved in maintaining uterine quiescence during pregnancy and progesterone withdrawal is involved in the initiation of labour. In addition, progesterone

potentiates the action of some tocolytic agents.

4. There is insufficient evidence on the benefits and harms of vaginal progesterone therapy given to women who have had a previous preterm birth. Our Cochrane review highlights that there is no information reported from randomised controlled trials on vaginal progesterone in reducing respiratory distress syndrome and other outcomes of relevance for women and their infants.

5. The benefits and harms of vaginal progesterone have been under investigated to date.

**RESEARCH PLAN**

**Study Design:**

Multicentred randomised, double blind, placebo-controlled trial.

**Inclusion Criteria:**

Pregnant women with a live singleton or twins confirmed at the time of trial entry between 18 and 23^6^ weeks gestation, who have a history of prior preterm birth at less than 37 weeks gestation and greater than 20 weeks gestation (either vaginal birth or caesarean birth) in the immediately preceding pregnancy, where the onset of labour occurred spontaneously, or in association with cervical incompetence, or following preterm prelabour ruptured membranes. Women who receive progesterone therapy early for early pregnancy support (prior to 16 weeks gestation) in the current pregnancy will be eligible for trial inclusion.

**Exclusion Criteria:**

Women with the following will not be eligible for participation:

- Women whose immediately *preceding* preterm birth at less than 37 weeks gestation was associated with:
  - Placental abruption or placenta praevia
  - Multiple pregnancy
  - Iatrogenic decision for early birth (for example related to fetal distress, preeclampsia, eclampsia)
- Women whose *current* pregnancy is associated with:
  - Active vaginal bleeding requiring hospital admission after 17^+6^ weeks of gestation
  - Current preterm prelabour ruptured membranes diagnosed prior to trial entry
  - Active labour (defined as the presence of uterine activity and cervical dilatation greater than 3cm)
  - Triplet or higher order multiple pregnancy
  - Known lethal fetal anomaly
  - Fetal demise
  - Progesterone treatment during the current pregnancy after 16 weeks gestation
  - Any contraindication to continuation of the pregnancy (eg. chorioamnionitis requiring delivery)
  - Any contraindication to progesterone therapy (known active liver disease; active or hormone-related thrombophlebitis or thromboembolic disorder; known or suspected breast or genital malignancy).

The use of progesterone in the following conditions is not contraindicated but the manufacturers recommend use with monitoring. These include serious depression and medical conditions that may be aggravated by fluid retention (asthma, epilepsy, migraine, known cardiac dysfunction, known renal dysfunction).

**Trial Entry:**

Eligible women will be identified in the antenatal clinic, given the trial information sheet and counselled by the researcher, before obtaining informed written consent. Randomisation will occur between 18 and 23^+6^ weeks gestation by telephoning the randomisation service of the Maternal Perinatal Clinical Trials Unit, Department of Obstetrics and Gynaecology, The University of Adelaide, on (08) 8161 7661. During the short telephone call, information will be given to check eligibility, describe the characteristics of the woman, enable stratification at randomisation so that similar types of women are allocated to the treatment groups and to assist in follow-up. Additional information will be obtained regarding baseline demographic characteristics and previous birth outcomes. The randomisation schedule will use balanced variable blocks, and will be prepared by an investigator not involved with recruitment or clinical care. There will be stratification of women according to plurality of the pregnancy (singleton versus twin pregnancy) and centre. Eligible women will be randomised to either vaginal progesterone therapy or vaginal placebo.

**Study Medication & Treatment Schedules:**

After randomisation, the woman will be allocated a study number that corresponds with the same number on her treatment pack. The woman, her caregivers and research staff assessing the trial outcomes will be blinded to treatment allocation. Treatment packs will appear identical for the treatment groups.

Treatment packs will contain 14 week’s supply of progesterone therapy, or similar appearing vaginal placebo therapy. Women will be asked to self-administer equivalent of 100mg vaginal progesterone each evening from 20 weeks gestation, or randomisation (if this occurs after 20 weeks gestation) until birth or 34 weeks gestation (whichever occurs first).

Women who develop prelabour ruptured membranes after trial entry will remain in their treatment groups for the purposes of analysis, but will be advised to discontinue using vaginal pessaries to reduce the risk of introducing vaginal or ascending infection.

In the event of the development of serious depression or a medical condition that may be aggravated by fluid retention (asthma, epilepsy, migraine, known cardiac dysfunction, known renal dysfunction), the clinician, at his/her discretion, may advise the woman to cease using the trial medication if he/she feels it would be in the woman’s best interests to do so. These women will also remain in their treatment groups for the purposes of analysis.

Vaginal progesterone preparations are available in Australia, in a dose that is consistent with the only randomised trial to date assessing vaginal progesterone (da Fonseca 2003).

**Follow-up of women in both treatment groups**

Women will be reviewed in the antenatal clinic according to the recommendation of the practitioner responsible for their care. At 34 weeks gestation, women will be asked about the occurrence of any side effects experienced and compliance with the treatment protocol. After birth, information will be obtained relating to birth and infant outcomes from the woman’s and infant’s case notes by the research assistant and the delivery form completed. Similarly, the postnatal and neonatal forms will be completed for each live born infant after discharge from hospital. Data collection forms will be checked and signed by the local clinical coordinator, and the information returned to the coordinating centre in Adelaide.

It is recognised that, at a later date, assessment of longer-term maternal and childhood outcomes following prenatal progesterone treatment will be important. As part of this follow-up initially women will be asked to complete questionnaires at four months postpartum relating to quality of life, anxiety, postnatal depression (as measured using the SF36 Health Survey Questionnaire (Marteau 1992), Edinburgh Depression Scale (Cox 1987), and Short Form Speilberger State Trait Inventory (Ware 1992), and preference for treatment and satisfaction with care and infant development (Bricker 1995). A further funding application will address later childhood development.

**Primary Study Endpoints**

The primary study outcome is:

• **Neonatal Respiratory Distress Syndrome** (defined by the incidence (increasing respiratory distress or oxygen requirement in a term or preterm infant requiring respiratory support) and severity of neonatal respiratory disease (mild=mean airway pressure (MAP) <7cm H_2_O, and/or fractional inspired oxygen (FiO_2_)<0.4; moderate=MAP 7-9.9cm H_2_O, and/or FiO_2_ 0.40-0.79; severe=MAP >10cm H_2_O, and/or FiO_2_ > 0.80 with need for ventilation) (Neonatal Network, 2004).

**Secondary Study Endpoints**

The secondary study endpoints are:

**1) Other respiratory outcomes** defined as: main respiratory diagnosis (that is, the main indication for respiratory support for the baby), need for and duration of oxygen therapy (including highest FiO_2_ (%) within 12 hours birth), and need for and duration of mechanical ventilation (including maximum peak pressure (cm H_2_O) within 12 hours birth), air leak syndrome, need for surfactant therapy, nitric oxide for respiratory support, and chronic lung disease (defined as the need for any respiratory support, supplemental oxygen or intermittent positive pressure ventilation or continuous positive airways pressure for a chronic pulmonary disorder on the day the baby reached 36 weeks’ postmenstrual age, for infants born before 32 weeks gestation, or continued oxygen requirement at 28 days of age for infants born after 36 weeks gestation).

**2) Adverse outcomes for the infant** defined as one or more of the following: preterm birth (defined as birth at less than 37 weeks gestation) and mortality (defined as either a stillbirth (intrauterine fetal death after trial entry and prior to birth), or infant death (death of a live born infant prior to hospital discharge, and excluding lethal congenital anomalies)); Apgar score <4 at 5 minutes of age; birth weight less than the 3rd centile for gestational age at birth and infant sex; cord pH <7.18; intraventricular haemorrhage on early cranial ultrasound; periventricular leucomalacia on later cranial ultrasound; inotropic support for the treatment of patent ductus arteriosus; proven necrotising enterocolitis; proven systemic infection within 48 hours of birth and treated with antibiotics; retinopathy of prematurity.

**3) Adverse outcomes for the woman** including length of antenatal hospital stay; use of tocolytic therapy; antenatal corticosteroid therapy; side effects of progesterone supplementation (including headache, nausea, breast tenderness, coughing); antepartum haemorrhage; pre-eclampsia; preterm prelabour ruptured membranes; prelabour ruptured membranes at or near term (defined as prelabour ruptured membranes after 36 weeks gestation); chorioamnionitis requiring antibiotic use during labour; postpartum haemorrhage; antibiotic use after birth; length of postnatal hospital stay; maternal death.

**Sample Size**

The clinical endpoint of respiratory distress syndrome has been chosen as the primary endpoint. For women eligible for this trial, the best estimate of the incidence of respiratory distress syndrome is 15%. This estimate is taken from the largest and most recent randomised controlled trial with a similar eligibility profile as planned in this trial (Meis 2003). Data from the trial suggests that progesterone therapy supplementation will reduce this risk of respiratory distress syndrome by 47%. Taking a more conservative reduction, a sample size of 984 women will be able to show a 40% reduction in respiratory distress syndrome from 15% to 9% (5% level of significance, two-tailed alpha, 80% power).

**Committee Structure**

The trial will have a multidisciplinary steering group, and an independent data monitoring committee with established terms of reference will be formed. A multidisciplinary adverse events committee blinded to treatment allocation will review the cause of death for all maternal and infant deaths. These data will be made available to the independent Data Monitoring Committee. Publications related to the trial will be prepared by the writing committee.

**Analysis and Reporting of Results**

The initial analysis will examine baseline characteristics of all randomised women, as an indication of comparable treatment groups, and include maternal age, race, height, weight, smoking history, past obstetric history (including previous preterm birth or perinatal loss), and reason for previous preterm birth.

Outcome comparisons will initially be made among all women and infants for the primary and secondary outcomes on an “intention to treat” basis, according to treatment allocation at randomisation to either progesterone or placebo. Subsequent analyses will examine the effects of gestational age for the previous preterm birth, the reason for the previous preterm birth, risk factors for preterm birth in the current pregnancy, and current single or multiple pregnancy. The relative risks and 95% confidence intervals will be reported for the major outcomes, and the number needed to treat to prevent one adverse outcome will be calculated. Regression techniques will be used to examine the influence of prognostic factors on the major outcomes.

**Confidentiality and Data Security**

Information relating to the trial will be kept in a locked secure filing cabinet, and on password protected database, accessible only to members of the coordinating committee.

**OUTCOME AND SIGNIFICANCE**

Respiratory disease as a consequence of preterm birth is the major cause of early neonatal mortality and morbidity. In Australia, over 7% of all births occur at a gestational age of less than 37 weeks, but account for in excess of 70% of perinatal mortality. Current prevention strategies have been ineffective in changing the overall rate of preterm birth.

Progesterone is involved in maintaining uterine quiescence during pregnancy and progesterone withdrawal associated with the initiation of labour. It is biologically plausible that progesterone supplementation may prevent preterm birth.

Systematic review of the limited randomised controlled trials reported suggests a benefit in progesterone supplementation for women at risk of preterm birth in prolongation of gestation. However, there has been only one small trial assessing the use of vaginal progesterone, with no infant outcomes reported. Clearly, high quality trials are a priority.

If vaginal progesterone therapy during pregnancy for women who have had a previous spontaneous preterm birth is an effective way of reducing the risk of respiratory distress syndrome associated with preterm birth, this would be a **highly beneficial, cost effective and worthwhile treatment to reduce the burden of a major cause of infant and childhood morbidity in being born too soon.**

**REFERENCES**

Abate A et al 17 hydroxyprogesterone in ART. Clin Exp Obst Gyn 1997;24:190.

Adams M et al Rates and factors associated with recurrence of preterm delivery. JAMA 2000;283(12):1591-1596.

ACOG Opinion: Progesteorne for preterm birth. Obstet Gynecol 2003;102:1115.

Astle S Progesterone in the onset of labour. EurJObstGynRepBiol 2003;108:177.

Australian collaborative trial of antenatal thyrotropin-releasing hormone (ACTOBAT) for prevention of neonatal respiratory disease. Lancet 1995;345:877.

Australia's mothers and babies 2000. Sydney: AIHW NPSU; 2003.

Bakketeig L et al. The tendency to repeat gestational age and birth weight in successive births. Am J Obstet Gynecol 1979;135(8):1086-1103.

Bloom S et al Recurrence of preterm birth. Obstet Gynecol 2001;98:379-385.

Bricker D et al Ages and stages questionnaire Brookes Publ. Co. USA; 1995.

Challis J et al Endocrine regulation of birth. End Rev 2000;21(5):514.

Challis J et al Prostaglandins & preterm birth. Reproduction 2002;124(1).

Cicinelli E et al Progesterone plasma levels. Gynecol Obstet Invest 1993;35:172.

Condon J et al A decline in the levels of progesterone receptor may antagonize progesterone receptor function Proc Natl Acad Sci USA 2003;100(16):9518-9523.

Cox J et al Detection of postnatal depression - development of the 10 item Edinburgh Postnatal Depression Scale (EDPS). Brit J Psych 1987;154:782-786.

Critchley H et al Preterm Birth. London: RCOG Publications; 2004.

Crossley K Suppression of arousal by progesterone. Rep Fert Dev 1997;9(8):767.

Crowley P. Prophylactic corticosteroids for preterm birth (Systematic Review).

The Cochrane Library 2006 Issue 1 Chichester UK, John Wiley & Sons Ltd.

Csapo AI. Progesterone "block". Am J Anat 1956;98:273-292.

da Fonseca E at al Prophylactic administration of progesterone by vaginal suppository: a randomized study. Am J Obstet Gynecol 2003;188:419-424.

deZiegler D et al Contractility of uterus. Ann NY Acad Sci 2001;943:172.

Dodd JM et al Prenatal administration of progesterone for preventing preterm birth (Review). The Cochrane Library 2006 Issue 1 Chichester UK, John Wiley & Sons Ltd.

The Cochrane Library 2006 Issue 1 Chichester UK, John Wiley & Sons Ltd.

Doggrell S Hope for preterm delivery. Exp Opin Pharmacother 2003;4(12):2363.

Donoghue D et al The report of the Australian and New Zealand Neonatal Network, 2000. Sydney: ANZNN; 2002.

Drummond M et al Methods for economic evaluation of health care OUP 1997.

Elder D Hospital admission in first year. J Paediatr Child Health 1999;35(2):145.

el-Refaey H, et al Medical management of missed abortion. BMJ 1992;305:1399.

Gibb W et al Mechanisms of birth. J Obstet Gynaecol Can 2002;24(11):874.

Grazzini E et al Inhibition of oxytocin receptor function by direct binding of progesterone. Nature 1998;392(6675):509-512.

Greenhlagh T. Papers that tell you what things cost. BMJ 1997;315:59.

Haluska G et al Progesterone receptor localization and isoforms in myometrium: evidence functional progesterone withdrawal. J Soc Gynecol Investig 2002;9(3):125.

Hartikainen-Sorri A Inefficacy of progesterone in twins. Obstet Gyn 1980;56:692.

Hauth J et al The effect of 17 alpha hydroxyprogesterone caproate on pregnancy outcome in an active-duty military population. Am J Obstet Gynecol 1983;146:187.

Hewitt BC, Newnham JP. A review of the obstetric and medical complications leading to the delivery of very low birth weight infants. MJA 1988;149:234-237.

Jenkin G, Thorburn G Inhibition of progesterone secretion by a 3 beta hydroxysteroid

dehydrogenase inhibitor. Can J Physiol Pharmacol 1985;63(2):136.

Johnson J et al Efficacy of progesterone in premature labor. NEJM 1975;293:675.

Kiss H et al Prospective randomised controlled trial of an infection screening programme to reduce the rate of preterm delivery. BMJ 2004;329:371.

Kramer M et al Contribution of preterm birth to mortality. JAMA 2000;284:843.

Laws P Australia's mothers and babies 2002. Sydney: AIHW, NPSU; 2004.

LeVine L. Habitual abortion. West J Surg 1964;72:30.

Levy T et al. Pharmacokinetics of natural progesterone. Hum Rep 1999;14(3):606.

Lopez Bernal A. Mechanisms of labour BJOG 2003;110(Suppl 20):39-45.

Marteau T Spielberger Trait Anxiety Inventory. BrJClinPsychol 1992;31:301.

McDonald H et al Antibiotics for treating bacterial vaginosis in pregnancy (Review). The Cochrane Library 2005 Issue 1 Chichester UK, John Wiley & Sons Ltd.

McLaughlin K et al. Who remains undelivered more than seven days after a single course of prenatal corticosteroids? Aust N Z J Obstet Gynaecol 2002;42(4):353-357.

Meis P et al Prevention of recurrent preterm delivery by 17-alpha hydroxyprogesterone caproate. NEJM 2003;348:2379-2385.

Mercer B et al The preterm prediction study: effect of gestational age and cause of preterm birth on obstetric outcomes. Am J Obstet Gynecol 1999;181(5):1216.

Mesiano S. Myometrial Progesterone Responsiveness and the Control of Human Parturition. J Soc Gynecol Investig 2004;11:193-202.

Mircioio C et al Pharmacokinetics of progesterone in postmenopausal women following vaginal administration. Eur J Drug Metab Pharmacokinet 1998;23(3):391.

Morrison JC. Preterm birth: a puzzle worth solving. Obstet Gynecol 1990;76(1):5S.

Nicol M et al Effect of alteration of maternal plasma progesterone concentrations on fetal behavioural state suring late gestation. J Endocrinol 1997;152(3):379-386.

Nicol M et al Effects of pregnanolone on behavioural parameters and responses to GABA(A) receptor antagonists in fetal sheep. Neuropharmacology 1999;38(1):49-63.

Norwitz E et al Defining standards in MFM. Am J Obstet Gynecol 2004;191:1491

Nyboe-Andersen A et al. Progesterone supplementation during IVF or ICSI: a randomized controlled trial. Human Reproduction 2002;17(2):357-361.

Onsrud M et al Intramuscular administration of hydroxyprogesterone caproate in endometrial carcinoma. Acta Obstet Gynecol Scand 1985;64(6):519-523.

Papiernik-Berkhauer E Etude en double aveugle d'un medicament prevenant la survenue prematuree de l'accouchement. Edition Schering Serie IV 3:65-68.

Pepe G et al Actions of placental and fetal hormones. End Rev 1995;16(5):608.

Penzias AS. Luteal phase support. Fertility and Sterility 2002;77(2):318-323.

Petrini J et al. Estimated effect of 17 alpha hydroxyprogesterone caproate on preterm birth in the United States. Obstetrics and Gynecology 2005;105:267-272.

Pieber D et al. Interactions between progesterone receptor isoforms in myometrial cells in human labour. Mol Hum Reprod 2001;7(9):875-879.

Raman L et al Fetal effects sex hormone exposure. Obstet Gynecol 1995;85:141.

Rodger M Abortion in early pregnancy with mifepristone. Lancet 1987;2:1415.

Schardein J Congenital abnormalities and hormones Teratology 1980;22:251.

Smit D et al Predictive value of uterine contractility and the serum levels of progesterone and oestrogens. Gynecol Obstet Invest 1984;18(5):252-263.

Smith R et al. Control of the length of gestation. In: Critchley H, Bennett P, Thornton S eds. Preterm Birth. London: RCOG Publications; 2004.

Smith R et al Hormone trajectories leading to birth. Reg Peptides 2002;108:159.

Stanley F Survival & cerebral palsy in infants. Paed Perinat Epid 1992;6(2):298.

Tavaniotou A Routes of progesterone administration. Hum Rep 2000;6(2):139.

Ware J 36 item short form health survey (SF36). Medical Care 1992;30:473-483.

Yemini M Prevent premature labor by progesterone. AmJObstGyn 1985;151:574.
